# Progress in Core–Shell Magnetic Mesoporous Materials for Enriching Post-Translationally Modified Peptides

**DOI:** 10.3390/jfb15060158

**Published:** 2024-06-06

**Authors:** Zhenyu Zhu, Hang Fu, Yu Zhao, Qiulin Yan

**Affiliations:** 1 Isotopomics in Chemical Biology (ICB), College of Chemistry & Chemical Engineering, Shaanxi University of Science and Technology, Xi’an 710021, China; fuhang@sust.edu.cn (H.F.); zhaoyu@sust.edu.cn (Y.Z.); yanqiulin@sust.edu.cn (Q.Y.); 2 Shaanxi Key Laboratory of Chemical Additives for Industry, Shaanxi University of Science and Technology, Weiyang University Park, Xi’an 710021, China

**Keywords:** core–shell structure, enrichment, magnetic mesoporous materials, post-translational modification, endogenous peptides

## Abstract

Endogenous peptides, particularly those with post-translational modifications, are increasingly being studied as biomarkers for diagnosing various diseases. However, they are weakly ionizable, have a low abundance in biological samples, and may be interfered with by high levels of proteins, peptides, and other macromolecular impurities, resulting in a high limit of detection and insufficient amounts of post-translationally modified peptides in real biological samples to be examined. Therefore, separation and enrichment are necessary before analyzing these biomarkers using mass spectrometry. Mesoporous materials have regular adjustable pores that can eliminate large proteins and impurities, and their large specific surface area can bind more target peptides, but this may result in the partial loss or destruction of target peptides during centrifugal separation. On the other hand, magnetic mesoporous materials can be used to separate the target using an external magnetic field, which improves the separation efficiency and yield. Core–shell magnetic mesoporous materials are widely utilized for peptide separation and enrichment due to their biocompatibility, efficient enrichment capability, and excellent recoverability. This paper provides a review of the latest progress in core–shell magnetic mesoporous materials for enriching glycopeptides and phosphopeptides and compares their enrichment performance with different types of functionalization methods.

## 1. Introduction

Peptides are biological macromolecules formed by the dehydration condensation of amino acids. They serve as the basic unit of protein composition and play a crucial role in regulating various physiological processes in organisms [1]. Additionally, peptides are involved in mineral binding [2,3], antibacterial activity [4], antihypertensive effects [5,6], antithrombotic activity [7], and antioxidant activity [8,9]. For instance, angiotensin II is an 8-peptide that increases blood pressure by narrowing small arteries throughout the body. Inhibiting angiotensin, however, can reduce blood pressure [5]. Post-translationally modified peptides and proteins, such as phosphorylated or glycosylated [10] peptides and proteins, play important roles in intercellular signaling [11] and neuromodulation [12]. However, abnormal post-translationally modified peptides and proteins may cause diseases such as Alzheimer’s disease [13], cancer [14], and cardiovascular disease [15]. Endogenous post-translationally modified peptides and proteins have been shown to be potential biomarkers of human physiology and pathology. Therefore, in-depth studies of these peptides can help understand the pathology of human disease and provide reliable information for disease diagnosis.

In recent years, the development of fast-scanning high-resolution mass spectrometry (HRMS) has provided a more reliable, efficient, and rapid method for analyzing small-molecule peptides [16]. This technology has become increasingly popular due to its ability to accurately identify and quantify compounds in complex samples. However, the concentration of endogenous peptides in real biological samples is extremely low, making them difficult to ionize. Additionally, they are often susceptible to interference from high-abundance macromolecular proteins, buffer salts, and surface-active substances introduced by pretreatment [17]. Furthermore, post-translationally modified endogenous peptides are dynamic in nature [18,19], which greatly limits their analysis by means of mass spectrometry. Therefore, it is necessary to selectively enrich low-abundance peptides and post-translationally modified peptides before mass spectrometry analysis [20].

General methods for separating and enriching post-translationally modified peptides include membrane separation, organic solvent precipitation, molecular imprinting and magnetic solid-phase extraction (MSPE). The membrane separation method is based on the selective transport of membranes to achieve the separation and enrichment of different components [21]. Meanwhile, the organic solvent precipitation method utilizes organic solvents to reduce the dielectric constant of water, resulting in a decrease in hydrophobic interactions between macromolecular proteins and an increase in electrostatic interactions, leading to aggregation and precipitation; the post-translationally modified peptides are then dissolved in organic solvent [22]. Molecular imprinting involves pre-polymerization of a template molecule with a functional monomer using a cross-linking agent. Removal of the template molecule results in a molecularly imprinted polymer that can specifically recognize and selectively bind to a target molecule [23]. The MSPE separates the target from interfering substances by exploiting the difference in force strengths between them on the extractant [24]. Membrane separation has the limitations of easy clogging and unstable recovery. The molecular weight range of target peptides obtained by organic solvent precipitation is unclear, and some target peptides may be co-precipitated with proteins. Molecular imprinting, on the other hand, has the advantage of high specificity, but it is not suitable for global analysis of endogenous peptides. MSPE has become the most commonly applied method for enriching endogenous peptides due to its simple operation, high extraction efficiency, low organic solvent consumption, and easy surface functionalization [24]. This method allows for the efficient separation of the magnetic adsorbent and the target peptide from the sample solution using an external magnetic field. The target peptide can then be obtained through elution, thus avoiding loss and denaturation caused by centrifugation. Some of the target peptides may co-precipitate with proteins, which represents a significant cause of poor recovery during centrifugation. One study demonstrated that peptide recoveries obtained by ultrafiltration and organic solvent precipitation were approximately half of those obtained by solid-phase extraction [25]. Magnetic materials such as iron (Fe), cobalt (Co), nickel (Ni), and their alloys are known to be biotoxic. Magnetite (Fe_3_O_4_) has good biocompatibility [26] and superb dispersibility in aqueous solutions and can reach adsorption equilibrium quickly. The magnetic properties of Fe_3_O_4_ are generated by the spin magnetic moments of Fe^2+^ and Fe^3+^ ions. The reduction in the Fe_3_O_4_ particle size from bulk to nanoscale reduces the number of exchange-coupled spins that spontaneously resist magnetic reorientation, a phenomenon that leads to superparamagnetic behavior. Furthermore, an increase in temperature also leads to superparamagnetic behavior [27]. It should be noted that under extreme conditions, Fe_3_O_4_ magnetic nanomaterials (Fe_3_O_4_ MNPs) may be less stable [28], which limits the application of this advanced material. A composite material with a magnetic core and a mesoporous shell exhibits both an improved stability of Fe_3_O_4_ MNPs and strong magnetic properties. Mesoporous materials are a type of porous materials with pore sizes ranging from 2 to 50 nm. They can effectively filter out large proteins and other bulky impurities in samples [29]. These materials have several advantages, including a large specific surface area, adjustable pore sizes, modifiable inner walls, and good stability. As a result, they are highly suitable for immobilizing and separating enzymes, proteins, and small-molecule peptides [30]. Fe_3_O_4_ belongs to spinel nanomaterials, which exhibit, among other characteristics, engineering defects that can be useful for tuning these kinds of materials for functionalization [31]. Fe_3_O_4_ MNPs have numerous hydroxyl functional groups on their surface [32], which are easily modifiable and covalently connected to the mesoporous shell. This shell not only provides mechanical strength but also enhances the stability of the Fe_3_O_4_ MNPs wrapped in it, making them highly promising for the enrichment of low-abundance peptides [33,34,35].

Mesoporous silica is a material with highly ordered pores, an easy functionalized surface, and good biocompatibility. Covalent organic frameworks (COFs) are porous crystalline materials consisting of organic monomers connected by covalent bonds with two- or three-dimensional structures. They have a low density, a controllable pore size, good stability, easy surface modification, and a high surface area. COFs have broad potential for adsorbing and enriching low-abundance post-translationally modified peptides [36]. Metal–organic frameworks (MOFs) are porous materials made up of metal ions/clusters and organic ligands. They have similar surface properties to COFs. However, MOFs have a large number of unsaturated metal sites, making them excellent adsorbent materials for enriching post-translationally modified peptides, especially phosphopeptides, in biological samples [37]. This paper presents a review of recent advances in the synthesis and modification strategies of core–shell magnetic mesoporous composites for enriching post-translationally modified peptides over the past five years. The composites discussed include magnetic mesoporous silica, magnetic COFs, and magnetic MOFs.

## 2. Physical and Chemical Properties of Core–Shell Magnetic Mesoporous Materials and Characterization Techniques

### 2.1. Physical Properties and Characterization Techniques of Core–Shell Magnetic Mesoporous Materials

The shape and size of core–shell magnetic mesoporous materials are typically quantified via transmission electron microscopy or swept electron microscopy. Iron oxide nanoparticles are categorized as ultra-small superparamagnetic iron oxides, superparamagnetic iron oxides, or micron-sized iron oxides according to their particle size, which is less than 50 nm, approximately 50–150 nm, or approximately 1 μm, respectively [38]. The magnetic properties of core–shell magnetic mesoporous materials are typically slightly inferior to those of Fe_3_O_4_MNPs. This is due to the fact that the outer layer of Fe_3_O_4_MNPs is encapsulated by a mesoporous shell, and their saturation magnetic values are generally determined using a vibrating sample magnetometer. The pore size of the mesoporous shell determines the molecular weight range of the adsorbed peptide, while the surface area and pore volume of the magnetic mesoporous material determine its ability to load peptides. The pore size, pore volume, and surface area of the magnetic mesoporous materials are determined by N_2_ adsorption–desorption isotherms [39].

### 2.2. Chemical Properties and Characterization Techniques of Core–Shell Magnetic Mesoporous Materials

In order to efficiently enrich post-translationally modified peptides, the surface of magnetic mesoporous materials is modified with compounds that can specifically bind to the post-translationally modified peptides. Infrared spectroscopy and Raman spectroscopy are based on spectral signals radiated by vibrations or rotations of chemical bonds and lattices, which can be used to determine the composition of the magnetic mesoporous material. These techniques are employed to ascertain whether the mesoporous shells and modified compounds are successfully bound to the magnetic cores [40]. X-ray photoelectron spectroscopy (XPS) can provide the elemental composition of the magnetic mesoporous material, thereby offering confirmatory information about the successful modification of the magnetic material [41]. X-ray diffraction (XRD) is employed to analyze the diffraction spectra of X-ray stimulated materials. Typically, a wide-angle XRD pattern indicates the presence of compound magnetic solids. A low-angle XRD pattern shows the pore structure of porous magnetic materials [42]. Thermogravimetric analysis is mainly used to determine the composition of the material and to predict its thermal stability. Nevertheless, when employing magnetic mesoporous materials to enrich endogenous peptides, which are frequently dispersed in solution, the zeta potential represents a means of characterizing the stability of the magnetic mesoporous material in solution. Zeta potential is the potential of colloidal particles moving under an electric field in the sliding/shear plane, and the greater the absolute value of the zeta potential, the more stable the system [43].

## 3. Progress in Magnetic Mesoporous Materials for Glycopeptide Enrichment—Strategies Based on Modification of Hydrophilic Compounds

Glycosylation is the process of attaching sugars to proteins through glycosyltransferases. It is the most common post-translational modification in the human body, accounting for about 50% of protein modifications [44]. The two main modes of glycosylation are O-linked and N-linked glycosylation. O-linked glycosylation mainly occurs on the hydroxyl groups of serine, threonine, hydroxylysine, and hydroxyproline. N-linked glycosylation mainly occurs on the amide group of aspartic acid, the α-amino group of N-terminal amino acids, and the ω-amino group of lysine or arginine. Glycosylation is a complex process that adds structural complexity to proteins. Identifying glycosylated proteins or peptides is a significant challenge. This subsection summarizes the advances and processes in the strategy of selective enrichment of glycopeptides based on functionalized magnetic mesoporous materials.

### 3.1. Magnetic Mesoporous Silica Materials for Glycopeptide Enrichment

Mesoporous silica is commonly used in composites coated with magnetic nanoparticles due to its high specific surface area, uniform and adjustable pores, easy surface functionalization, and good stability. Based on its size exclusion effect and good magnetism, the step of enriching low-abundance peptides and low-molecular-weight proteins from complex biological matrices becomes simple, fast, and efficient.

**Figure 1:**
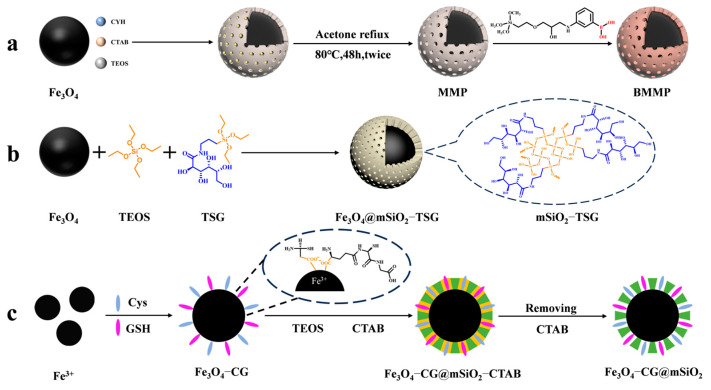
Magnetic mesoporous silica materials for enrichment of glycopeptides, the synthesis steps of BMMP, Fe_3_O_4_@mSiO_2_−TSG, and Fe_3_O_4_@mSiO_2_−TSG are represented by processes (**a**), (**b**), and (**c**), respectively.

In recent years, the enrichment and identification of peptides in organisms have shifted towards glycosylated peptides; as one of the most important post-translational modifications, glycopeptides carry rich biological information. A large number of studies have shown that glycopeptides carry specific diagnostic information for certain diseases, especially cancers. Thus, the study of glycopeptides can improve the methods for diagnosing diseases in humans. However, glycopeptides have a large spatial structure, and the small pore size (2~4 nm) of mesoporous silica in general does not allow some larger glycopeptides to enter. Yang et al. [45] designed a magnetic mesoporous silica material modified with boric acid (shown in Figure 1a). They expanded the pore size of the mesoporous layer to 10 nm in the presence of cyclohexane and functionalized the surface with boric acid to enhance the material’s hydrophilicity. The material achieved multimodal global enrichment of glycopeptides by utilizing the reversible binding of boric acid to cis-diols. Under alkaline conditions, boric acid can form a stable cyclic ester with cis-diols. This ester hydrolyzes in acidic environments, allowing for glycopeptide-specific binding and elution. Boric acid chemistry can yield intact glycopeptides, without altering their structures, but the different affinities between boric acid and different glycopeptide chains limit the generalizability of the method.

**Figure 2:**
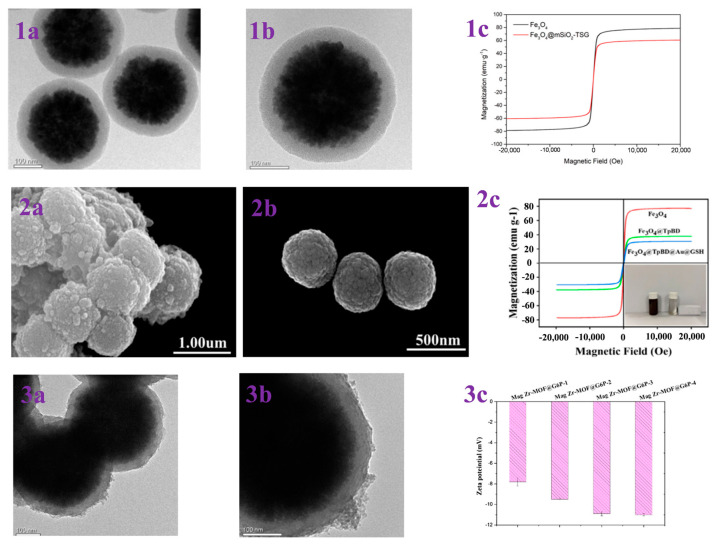
(**1a**,**1b**) TEM images of Fe_3_O_4_@mSiO_2_-TSG; (**1c**) magnetic hysteresis curves of Fe_3_O_4_ and Fe_3_O_4_@mSiO_2_-TSG. Reprinted with permission from Ref. [47]. Copyright 2024 Elsevier. (**2a**,**2b**) SEM of Fe_3_O_4_@TpBD@Au@GSH and Fe_3_O_4_; (**2c**) VSM analysis of Fe_3_O_4_, Fe_3_O_4_@TpBD and Fe_3_O_4_@TpBD@Au@GSH. Reprinted with permission from Ref. [49]. Copyright 2024 Elsevier. (**3a**,**3b**) TEM images of Mag Zr-MOF@G6P; (**3c**) zeta potentials of Mag Zr-MOF@G6P−1/2/3/4. Reprinted with permission from Ref. [50]. Copyright 2024 Springer Nature.

In contrast, hydrophilic interaction chromatography (HILIC) has gained increasing attention due to its excellent enrichment ability and mild enrichment conditions. Glycopeptides possess strong hydrophilicity due to the polyhydroxyl structure of the glycosyl part, while non-glycopeptides are less hydrophilic. Based on these differences, HILIC can obtain the intact glycopeptide structure and enrich all types of glycopeptides without bias. Chen et al. [46] reported core–shell magnetic mesoporous silica microspheres modified by L-cysteine, denoted as L-Cys-Fe_3_O_4_@mSiO_2_. A large amount of L-cysteine was modified within the vertically aligned apertures utilizing Fe-S bonding. This modification improved the hydrophilicity of the material, enabling it to have a strong recognition and adsorption ability. While the hydrophilicity of the material can enhance the efficiency of glycopeptide enrichment, the process of functionalizing the modification on the surface of core–shell magnetic nanomaterials is typically cumbersome. Xu et al. [47] reported a one-step synthesis of strongly hydrophilic magnetic mesoporous silica nanomaterials modified with N-(3-triethoxysilylpropyl) lucosamido-amido (TSG), denoted as Fe_3_O_4_@mSiO_2_-TSG (shown in Figure 1b and Figure 2(1a–1c)). The sol–gel method was used to carry out this procedure, with tetraethyl orthosilicate (TEOS) and TSG serving as the silica source and CTAB as the surfactant templating agent. The TSG-functionalized silica layer was then deposited on Fe_3_O_4_ spheres, and then the CTAB was removed through heating and refluxing to obtain Fe_3_O_4_@mSiO_2_-TSG. The synthesis steps were greatly simplified by this method. The detection limit for the selective enrichment of glycopeptides in the HRP digests was 0.1 f·mol·μL^−1^, and the maximum adsorption capacity was 160 mg/g. The material can be reused up to eight times. Even when the molar ratio of glycoprotein ovalbumin to HRP digest was 1:5000, the mass spectra after enrichment were still dominated by glycopeptide peaks and protein peaks were not detected, indicating good selectivity. After enriching with Fe_3_O_4_@mSiO_2_-TSG, we identified 162 glycopeptides corresponding to 71 glycoproteins in healthy human serum. In serum from breast cancer patients, we identified 156 glycopeptides corresponding to 70 glycoproteins.

While the selectivity of HILIC is interfered with by hydrophilic peptides that do not contain glycans, leading to a decrease in selectivity for glycopeptides, HILIC is being developed in the direction of superhydrophilic materials with stronger hydrophilicity to improve its selection of glycopeptides. Yi et al. [48] designed a superhydrophilic magnetic mesoporous silica microspheres modified with reduced glutathione (GSH) and L−cysteine (Cys), named Fe_3_O_4_−CG@mSiO_2_ (shown in Figure 1c). Superhydrophilic Fe_3_O_4_−CG nanospheres were first synthesized by Fe-S interactions, and then a mesoporous silica shell was modified on the surface of Fe_3_O_4_−CG via the surfactant template method, with CTAB as a structure-directing agent to give it the property of specifically adsorbing glycopeptides by excluding large proteins. Fe_3_O_4_−CG@mSiO_2_ nanospheres were enriched with a high sensitivity and selectivity and could be reused ten times with an average recovery of 108.6 ± 5.5%. For the selective enrichment of glycopeptides in HRP digests, the detection limit was 5 a·mol·μL^−1^. BSA protein was used as an interfering agent to verify the selectivity of Fe_3_O_4_−CG@mSiO_2_ nanospheres, and when the molar ratio of BSA protein to HRP digest reached 1:10,000, Fe_3_O_4_-CG@mSiO_2_ nanospheres could still enrich seven glycopeptides. In addition, 156 glycopeptides from 64 human serum exosomal proteins were detected using Fe_3_O_4_−CG@mSiO_2_.

Functionalized magnetic mesoporous silica materials exhibit a high selectivity and adsorption capacity during the enrichment step before mass spectrometry characterization of glycopeptides. When modifying magnetic mesoporous materials, it is important to consider not only the hydrophilicity of the material, which is central to the HILIC strategy, but also the complexity of the material synthesis method and its cost.

### 3.2. Magnetic COF Materials for Glycopeptide Enrichment

**Figure 3:**
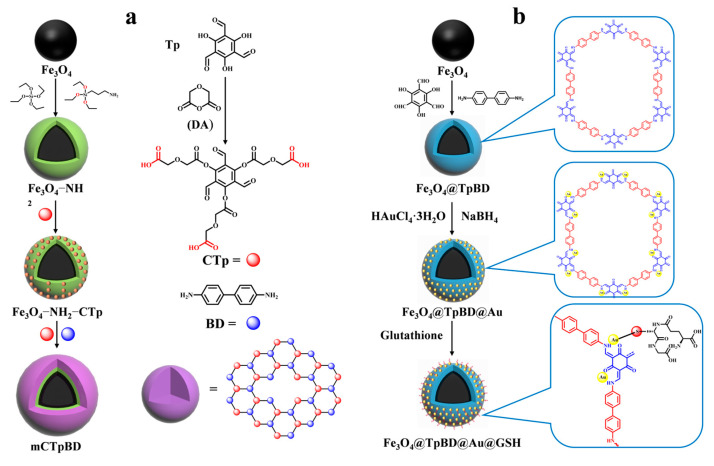
Magnetic covalent organic framework materials for enrichment of glycopeptides, the synthesis steps of mCpBD and Fe_3_O_4_@TpBD@Au@GSH are represented by processes (**a**) and (**b**), respectively.

Various modified covalent organic frameworks (COFs) [51] have been reported for peptide enrichment since the first successful synthesis of COFs. The following methods are employed in the preparation of magnetic COFs: the Schiff base condensation method, the template-controlled precipitation polymerization method, the co-precipitation method, the microwave-enhanced high-temperature ionization thermophoresis method, and the solvent thermophoresis method. Among the aforementioned methods, the template-controlled precipitation polymerization method employs carboxyl groups to uniformly disperse the magnetic nanoparticles within the synthesis system, thereby ensuring the formation of imide-based COFs on the surface of the magnetic nanoparticles. This method enables the synthesis of magnetic COFs with core–shell structures under mild conditions, obviating the necessity for cumbersome reaction steps. Furthermore, it represents a more economical and straightforward approach to the synthesis of magnetic COFs [52]. However, general COFs with a low hydrophilicity are less effective for enriching glycosylated peptides with a high hydrophilicity. Therefore, it is necessary to enhance the hydrophilicity of COFs to improve their enrichment efficiency of glycosylated peptides. There are two ways to achieve this. The first is modifying the functional groups with hydrophilic properties on the monomers of the synthesized COFs, as demonstrated by Wu et al. [53]. COF shells were synthesized using carboxyl-modified 1,3,5-tricarboxy-mesityltriphenol (CTp) and p-diaminobiphenyl as the monomers on amine-amidated Fe_3_O_4_ (as shown in Figure 3a). This resulted in the successful enrichment of glycopeptides from the saliva of both healthy individuals and patients with inflammatory bowel disease in magnetic COF hydrophilic composites (mCTpBD). The detection limit of mCTpBD for glycopeptides was as low as 0.5 f·mol·μL^−1^ (HRP standard protein digest) based on the modification of carboxyl groups. Additionally, 28, 32, and 49 glycopeptides were detected in three healthy human saliva samples, while 27, 39, and 40 glycopeptides were detected in three patient saliva samples. The particle size exclusion effect was 1/1000 (HRP digestion product/BSA protein, *w*/*w*).

While modifying hydrophilic functional groups on monomers may lead to the reaction of the introduced groups with other functional groups, causing a change in the structure of the covalent organic framework, an alternative method to improve the hydrophilicity of COFs is to modify the hydrophilic groups after synthesis. This approach avoids the aforementioned scenario. Su et al. [49] synthesized glutathione-modified dual hydrophilic magnetic COF composites (Fe_3_O_4_@TpBD@Au@GSH) using a two-step post-synthesis modification method with 2,4,6-trihydroxybenzene-1,3,5- tricarbaldehyde (Tp) and benzidine (BD) as monomers to form the COF layer (shown in Figure 2(2a–2c) and Figure 3b). Glutathione was then grafted onto the surface of TpBD using Au-S bonds. Due to the hydrophilicity of TpBD and the superhydrophilicity of glutathione, Fe_3_O_4_@TpBD@Au@GSH exhibited a low sensitivity of 0.1 f·mol·μL^−1^ for standard glycopeptides (HRP tryptophan digest), a maximum loading of 160 mg/g for glycopeptides, good immunity to interference from large proteins (1:2000, HRP tryptophan digest/bovine serum albumin, M/M), and an excellent selectivity (1:2000, HRP tryptic digest/bovine serum albumin tryptic digest, M/M). A total of 492 glycopeptides and 160 glycopeptides were detected in 5 μL of human serum and saliva, corresponding to 134 and 64 glycoproteins, respectively, which demonstrated an excellent performance in the enrichment of endogenous glycopeptides. However, the post-synthetic modification steps are relatively cumbersome and costly. Therefore, there is a need to develop simpler and more cost-effective synthetic methods for wider application.

### 3.3. Magnetic MOF Materials for Glycopeptide Enrichment

**Figure 4:**
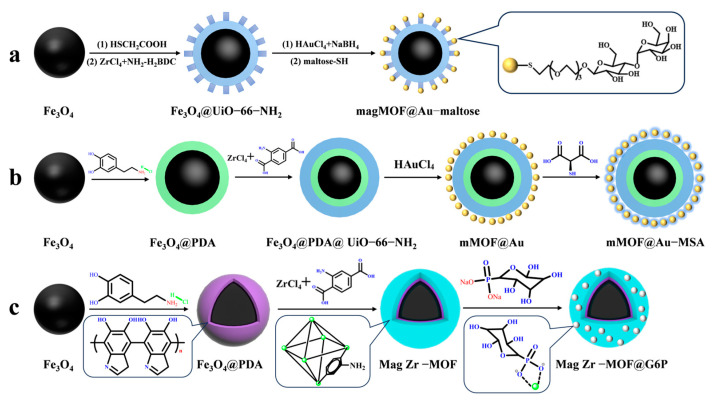
Magnetic metal–organic framework materials for enrichment of glycopeptides, the synthesis steps of magMOF@Au−maltose, mMOF@Au−MSA and Mag Zr−MOF@G6P are represented by processes (**a**), (**b**) and (**c**), respectively.

Magnetic MOF composites modified with hydrophilic molecules are widely used for enriching low-abundance glycopeptides due to their efficient enrichment performance, non-destructive separation, and excellent reproducibility. This makes them a suitable choice for glycopeptide enrichment. UiO-66-NH_2_ has been shown to be stable in humid, high-temperature, and acidic chemical environments, while also being biocompatible. A study by Lu et al. [54] supports these findings. Mag MOF@Au–maltose (shown in Figure 4a) was used to immobilize hydrophilic maltose onto magnetic MOF composites, with the Au-S bond serving as the connecting bond. This composite demonstrated an excellent glycopeptide enrichment performance, with a low detection limit of 10 f·mol, a high recovery rate of over 83.3%, and a large binding capacity of 83 μg/mg. In 1 μL of human serum, 113 glycopeptides with 123 unique N-glycosylation sites were detected, corresponding to 46 different glycoproteins.

Similarly, Hu et al. [55] used highly hydrophilic mercaptosuccinic acid (MSA) attached to the surface of magnetic UiO-66-NH_2_ modified with gold nanoparticles using a Au-S bond as a bridge to obtain mMOF@Au-MSA (shown in Figure 4b). The MOF’s large surface area provided a large number of binding sites for mercaptosuccinic acid, resulting in an enhanced detection limit of glycopeptides of 0.5 f·mol·μL^−1^ (HRP digest) and an increased enrichment capacity of 100 mg/g. In 2 μL of breast serum from breast cancer patients, 307 unique glycopeptides from 96 glycoproteins were detected. This may be due to the smaller spatial site resistance of mercaptosuccinic acid compared to maltose, which makes it more favorable for glycopeptides to enter the pores of MOFs. While noble metal nanoparticles have a tendency to aggregate, leading to clustering of functional groups and higher costs, which makes them impractical for many applications, this group [50] designed a hydrophilic glucose-6-phosphate (G6P)-modified magnetic UiO-66-NH_2_ material. Mag Zr-MOF@G6P was used to analyze N-glycopeptides in the urine of healthy people and kidney cancer patients by utilizing the coordination between Zr^4+^ and phosphate groups present in UiO-66-NH_2_ as a bridge. The structure of Mag Zr-MOF@G6P is shown in Figure 2(3a–3c) and Figure 4c. A total of 123 N-glycopeptides with 104 glycosylation sites and 111 N-glycopeptides with 97 glycosylation sites, corresponding to 78 and 76 glycoproteins, respectively, were detected from the urine of renal cancer patients and healthy subjects. These findings suggest that the detected glycoproteins are significantly associated with cancer bioprocesses. Table 1 presents the properties of the core-shell magnetic mesoporous materials discussed in the text, which are employed for the enrichment of glycopeptides.

Overall, the boronic-acid-method-based affinity strategy can obtain intact glycopeptides without destroying their structure. This is undoubtedly useful for exploiting the bioinformation carried by glycopeptides. However, its different affinities for different glycan chains limit its generalizability. Hydrophilic interaction chromatography (HILIC) enables the complete characterization of glycopeptide structures and provides highly specific enrichment of glycopeptides based on their hydrophilicity. Nevertheless, the HILIC strategy is not without its limitations, particularly in terms of achieving highly selective enrichment. For instance, some HILIC materials are capable of enriching neutral or positively charged glycopeptides, but are less effective at enriching negatively charged glycopeptides [56]. Additionally, the introduction of positively charged metal ions can enhance the binding ability of HILIC materials to negatively charged glycopeptides. However, it is important to note that HILIC materials may also bind some hydrophilic non-glycopeptides and other hydrophilic substances. HILIC can be combined with other strategies, such as lectins and IMAC, with the aim of increasing the density of hydrophilic groups on the immobilized material and thereby improving the detection sensitivity and loading capacity. Furthermore, the functional groups and metal ions present on the surface of COFs and MOFs provide numerous binding sites for hydrophilic molecules. Secondly, the structural diversity of COFs and MOFs allows for the exploration of additional functions, and self-assembled COFs and MOFs can be specifically modified for glycopeptides with varying properties. This characteristic allows for a wider range of applications in enriching glycopeptides, potentially leading to significant advancements in the field.

## 4. Progress in Magnetic Mesoporous Materials for Phosphopeptide Enrichment

Protein phosphorylation is mediated by kinases and phosphatases, which alter the protein conformation and charge distribution, thereby changing protein–protein interactions and enzyme activities. This process directs biological processes such as cell growth, division, differentiation, apoptosis, and signaling [57]. It occurs predominantly on serine and threonine residues. Reversible phosphorylation is a dynamic process [58] that occurs extensively in eukaryotic cells. Its occurrence is typically rare, and abnormal phosphorylation has been linked to certain diseases [59]. For instance, serum endogenous peptide levels have been proposed as biomarkers for cancer. Zhai et al. [60] employed zirconium arsenate-modified magnetic nanoparticles (ZrAs-Fe_3_O_4_@SiO_2_) to enrich and quantify four phosphopeptides in serum and observed that the levels of two of the phosphopeptides in the serum of colorectal patients were significantly reduced in comparison to healthy controls. The expression levels of these two phosphopeptides may represent the expression and activity of proteases, kinases, and phosphatases in individual serum, and maintain the dynamic balance of these enzymes. Therefore, it was possible to identify serum phosphopeptides as biomarkers for cancer. Direct detection of low-ionization phosphopeptides is not possible. Therefore, the study of methods to enrich and identify phosphopeptides has garnered significant attention. This subsection summarizes recent research progress on strategies for enriching phosphopeptides.

### 4.1. Magnetic Mesoporous Silica Materials for Phosphopeptide Enrichment

This enrichment is based on the chelation of metal ions with phosphate groups, following the immobilized metal ion affinity chromatography strategy (IMAC) and metal oxide affinity chromatography strategy (MOAC). The section describes the advances in the enrichment of phosphopeptides using magnetic mesoporous silica materials functionalized with metal ions and metal oxides.

#### 4.1.1. Strategies for Immobilized Metal ion Affinity Chromatography

**Figure 5:**
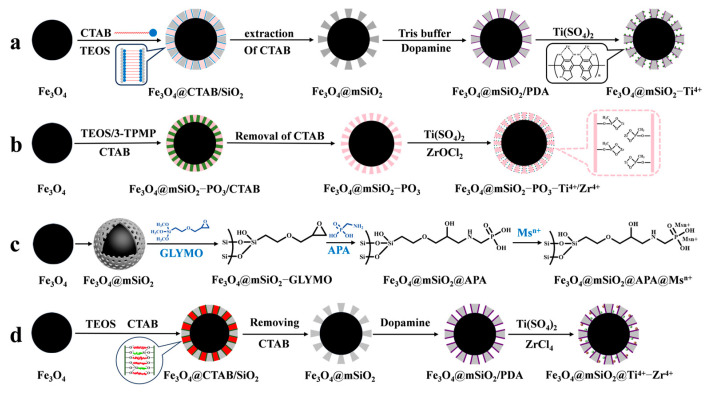
Magnetic mesoporous silica materials functionalized with metal ions, the synthesis steps of Fe_3_O_4_@mSiO_2_−Ti^4+^, Fe_3_O_4_@mSiO_2_−PO_3_−Ti^4+^/Zr^4+^, Fe_3_O_4_@mSiO_2_@APA@Ms^n+^ and Fe_3_O_4_@mSiO_2_@Ti^4+^−Zr^4+^ are represented by processes (**a**), (**b**), (**c**) and (**d**), respectively.

IMAC material is composed of three parts: metal ions for binding phosphopeptides, chelating ligands for immobilizing metal ions, and a matrix. The principle of enriching phosphopeptides with IMAC material is based on the ligand chelating between metal ions and phosphate groups. Different metal ions have varying affinities and coordination numbers for phosphate groups, so the selection of metal ions determines the efficiency of IMAC for phosphopeptide enrichment. As an example, Yao et al. [61] used polydopamine as a coupling agent to immobilize Ti^4+^ on the surface of magnetic mesoporous silica. This was conducted to enrich phosphopeptides in human serum and saliva before mass spectrometry analysis in order to reduce interference from biological macromolecules such as non-phosphopeptides (as shown in Figure 5a). Traditional metal ions, such as Cu^2+^, Fe^3+^, In^3+^, Ga^3+^, and Ti^4+^, have been extensively studied and are well established. However, their weak binding with phosphate groups results in the incomplete enrichment of phosphopeptides in organisms. To achieve a more comprehensive enrichment of phosphopeptides, alternative methods must be explored. Studies have shown that Ti^4+^, Ce^4+^, and Fe^3+^ have a greater tendency to enrich monophosphate peptides, while Nb^5+^, Zr^4+^, Ga^3+^, Y^3+^, and In^3+^ have a greater tendency to enrich polyphosphate peptides [62]. IMAC materials with single metal ions are not effective in comprehensively enriching and identifying phosphopeptides. It has been demonstrated that iron ions tend to bind acidic phosphopeptides, while titanium ions tend to bind basic phosphopeptides. Consequently, the enrichment of phosphopeptides in biological samples is not as comprehensive as might be expected from a single metal ion. However, the use of bimetallic ion MIAC material compensates for this [63]. In recent years, there has been a trend towards developing bimetallic IMAC materials that combine the advantages of different ions to achieve more comprehensive phosphopeptide enrichment. Fang et al. [64] introduced phosphates in the formation of mesoporous silica via a one-pot synthesis (as shown in Figure 5b). They then immobilized Ti^4+^ and Zr^4+^ on the magnetic mesoporous silica surface using phosphate, resulting in Fe_3_O_4_@mSiO_2_-PO_3_-Ti^4+^/Zr^4+^. The strong coordination between phosphate and metal ions was used as a chelating agent for the IMAC material, which greatly reduced the loss of metal ions. The (Fe_3_O_4_@mSiO_2_-PO_3_-Ti^4+^/Zr^4+^) material had a limit of detection of 1 f mol uL^−1^ (β-casein digest) and exhibited good selectivity for phosphopeptides (1:500, β-casein: BSA, m/m). In healthy saliva samples, 25 phosphopeptides were detected, while in gastritis saliva samples, 85 phosphopeptides were detected.

However, the chelating ligands utilized to immobilize metal ions can also impact the enrichment ability of IMAC materials. Common chelating ligands, such as iminodiacetic acid (IDA) and nitrilotriacetic acid (NTA), coordinate with metal ions through their carboxyl groups. However, this coordination is weak, leading to the loss of metal ions during elution of phosphopeptides under acidic conditions. Additionally, these ligands tend to bind with acidic peptides, reducing the selectivity for phosphopeptides. Liu et al. [65] developed a new chelating agent, aminomethylphosphonic acid, to bind Ti^4+^ and Nb^5+^ immobilized on a magnetic mesoporous silica matrix (Fe_3_O_4_@mSiO_2_@APA@Ti^4+^/Nb^5+^). This allowed for the comprehensive enrichment and identification of phosphopeptides in organisms, as shown in Figure 5c. Fe_3_O_4_@mSiO_2_@APA@Ti^4+^/Nb^5+^ exhibited a high sensitivity (0.05 f·mol·uL^−1^ β-casein digest) and good selectivity (β-casein to BSA mass ratio of 1:1500), as well as a high loading capacity of phosphopeptides (330 μg/mg). The polydopamine chelator, which has gained attention in recent years, possesses amino and hydroxyl bifunctional groups. This not only results in a strong chelating effect with metal ions but also provides good hydrophilicity, super adhesion, and biocompatibility. Hu et al. [66] modified magnetic mesoporous silica with metal ions Ti^4+^ and Zr^4+^ to obtain Fe_3_O_4_@mSiO_2_@Ti^4+^-Zr^4+^ affinity probes (shown in Figure 5d). These probes were used for the selective enrichment of endogenous phosphopeptides in human saliva. The mesoporous material with superparamagnetism, ordered mesoporous channels, and bimetallic ions affinity sites was obtained by first synthesizing Fe_3_O_4_@mSiO_2_ using the sol–gel method. Next, polydopamine was coated onto the surface of Fe_3_O_4_@mSiO_2_, and finally, Ti^4+^ and Zr^4+^ were grafted onto the polydopamine. The limit of detection was 0.1 f mol μL^−1^ for β-casein digest, and it was found to have a selectivity of 1:1000:1000 (β-casein digest/BAS protein/α-casein). The affinity probes, Fe_3_O_4_@mSiO_2_@Ti^4+^-Zr^4+^, were able to capture 95 single-phosphate peptides and 9 polyphosphate peptides from human saliva. The probes can be reused up to five times.

#### 4.1.2. Strategies for Metal Oxide Affinity Chromatography

**Figure 6:**
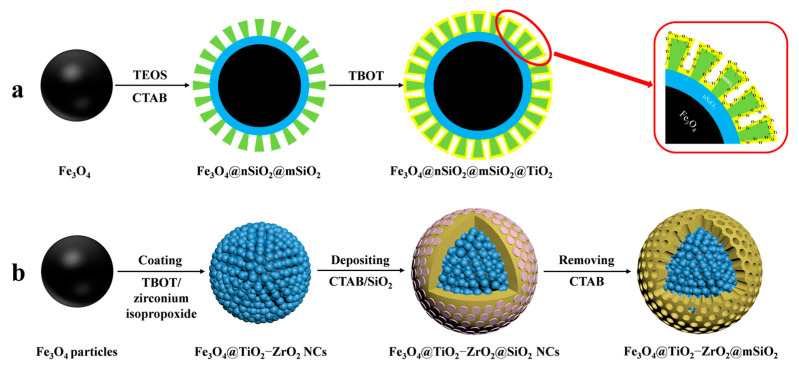
Magnetic mesoporous silica materials functionalized with metal oxide, the synthesis steps of Fe_3_O_4_@nSiO_2_@mSiO_2_@TiO_2_ and Fe_3_O_4_@TiO_2_−ZrO_2_@mSiO_2_ are represented by processes (**a**) and (**b**), respectively.

**Figure 7:**
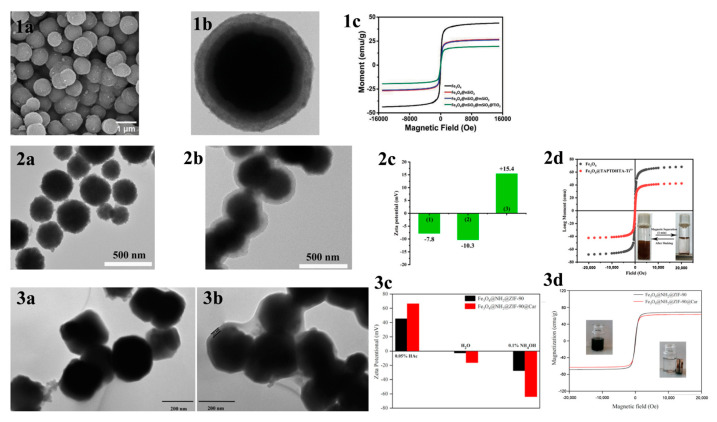
(**1a**) SEM and (**1b**) TEM images of Fe_3_O_4_@nSiO_2_@mSiO_2_@TiO_2_; (**1c**) magnetic hysteresis curves of Fe_3_O_4_, Fe_3_O_4_@nSiO_2_, Fe_3_O_4_@nSiO_2_@mSiO_2_ and Fe_3_O_4_@nSiO_2_@mSiO_2_@TiO_2_. Reprinted with permission from Ref. [68]. Copyright 2024 Elsevier. (**2a**,**2b**) TEM images of the Fe_3_O_4_ NPs and Fe_3_O_4_@TAPTDHTA composites, (**2c**) zeta potential of the (1) Fe_3_O_4_ NPs, (2) Fe_3_O_4_@TAPTDHTA composites and (3) Fe_3_O_4_@TAPTDHTA-Ti^4+^ composites, and (**2d**) magnetic hysteresis curve of the Fe_3_O_4_ NPs (black curve) and Fe_3_O_4_@TAPTDHTA-Ti^4+^ composites (red curve). Reprinted with permission from Ref. [70]. Copyright 2024 Elsevier. (**3a**,**3b**) TEM micrographs of Fe_3_O_4_@NH_2_@ZIF-90 and Fe_3_O_4_@NH_2_@ZIF-90@Car, (**3c**) zeta potential, and (**3d**) magnetic hysteresis curves. Reprinted with permission from Ref. [71]. Copyright 2024 Elsevier.

MOAC materials have an affinity for phosphopeptides due to the chelation of metal ions with phosphate groups. In acidic conditions, metal oxides bind to phosphate groups on peptides via bridging bidentate chelation. In contrast, under alkaline conditions, captured phosphopeptides elute via reversible Lewis acid–base interactions. The differing pKa values of various metal oxides result in different binding capacities of different metal oxides to phosphopeptides. The kinetic mechanism of the adsorption of phosphopeptides by metal oxides is not well understood due to the complex physicochemical properties of their surfaces [67]. In contrast to IMAC, MOAC materials immobilize metal ions on their surface in the form of oxides. This prevents the loss of metal ions that can occur in the IMAC method and allows for direct modification of the material surface without the need for chelating agents. Gao et al. [68] synthesized Fe_3_O_4_@nSiO_2_@mSiO_2_@TiO_2_, a titanium-dioxide-modified magnetic mesoporous material (shown in Figure 6a and Figure 7(1a–1c)). They also synthesized three mesoporous layers with different pore sizes (12.51 nm, 6.38 nm, and 1.82 nm) as a trypsin reactor by controlling the appropriate experimental conditions. The results indicate that the mesoporous layer with a pore size of 6.38 nm has a larger internal surface area and internal space, avoiding the shadowing effect caused by a pore size that is too small. This makes it a good temporary immobilized enzyme reactor, shortening the trypsin digestion process from 16 h to 2 h. Additionally, one-step selective enrichment of phosphopeptides was also established, allowing for protein digestion and phosphopeptide enrichment to be carried out in the same system, reducing the additional loss and interference of the sample. Fe_3_O_4_@nSiO_2_@mSiO_2_@TiO_2_ exhibits a high sensitivity (0.5 f·mol·μL^−1^ β-casein), good selectivity (molar ratio of β-casein: BAS of 1:2000), and high loading capacity (120 mg/g). In skimmed milk, 13 unique phosphopeptides were extracted, while in HepG2 cells, 1904 unique phosphopeptides were extracted. Additionally, a label-free quantitative proteomic analysis was conducted to examine phosphorylated proteins. The analysis was based on one-step selective enrichment of phosphopeptides. The results were obtained from mixed samples containing BAS and different concentrations of β-casein. The study found that the uniquely phosphorylated peptides of β-casein (m/z 2061.77) showed a linear correlation with the concentration of β-casein, with R = 0.9987.

Similar to IMAC, MOAC materials exhibit varying affinities of different metal oxides for phosphopeptides. TiO_2_ has a stronger affinity for polyphosphopeptides, while ZrO_2_ has a stronger affinity for monophosphopeptides. Binary bimetallic oxides of MOAC materials have attracted much attention for a more comprehensive identification of endogenous phosphopeptides. This was discussed by Li et al. [69]. Bimetallic-oxide-modified core–shell magnetic mesoporous materials (Fe_3_O_4_@TiO_2_-ZrO_2_@mSiO_2_) were synthesized and designed to enrich endogenous phosphopeptides in human saliva (shown in Figure 6b). The ability of these materials to enrich phosphopeptides was compared to that of the monometallic oxide composites Fe_3_O_4_@TiO_2_@mSiO_2_ and Fe_3_O_4_@ZrO_2_@mSiO_2_. Fe3O_4_@TiO_2_-ZrO_2_@mSiO_2_ detected 30 phosphopeptides in human saliva, while Fe_3_O_4_@TiO_2_@mSiO_2_ and Fe_3_O_4_@ZrO_2_@mSiO_2_ detected only 11 and 10 phosphopeptides.

Immobilized metal ion affinity chromatography (IMAC) and metal oxide affinity chromatography (MOAC) are highly selective and sensitive due to their affinity interactions between metal ions and metal oxides with the phosphate groups of phosphopeptides, resulting in specific binding of phosphopeptides. A Lewis acid–base reaction was the preferred method for MOAC enrichment of monophosphopeptides, while in IMAC, the interaction between metal ions and phosphate groups was favored for polyphosphopeptide enrichment.

### 4.2. Magnetic COF Materials for Phosphopeptide Enrichment

**Figure 8:**
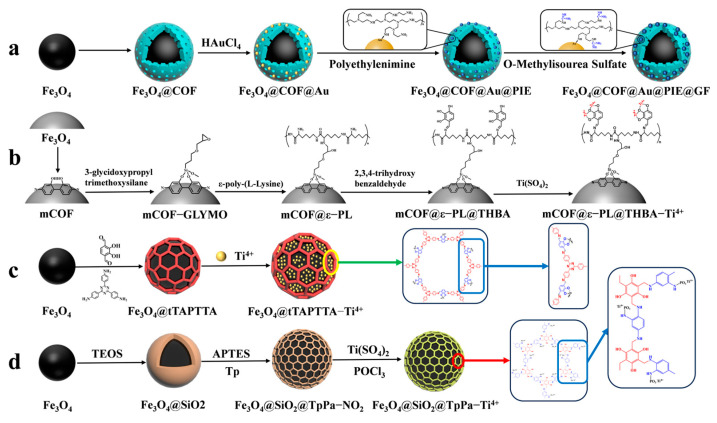
Magnetic covalent organic framework materials for enrichment of phosphopeptides, the synthesis steps of Fe_3_O_4_@COF@Au@PIE@GF, mCOF@ε−PL@THBA−Ti^4+^, Fe_3_O_4_@tTAPTTA−Ti^4+^ and Fe_3_O_4_@SiO_2_@TpPa−Ti^4+^ are represented by processes (**a**), (**b**), (**c**) and (**d**), respectively.

Guanidine-functionalized composites have recently been utilized to capture phosphopeptides in complex biological samples through non-covalent bonding interactions between guanidine and phosphate groups. Wang et al. [72] proposed a composite material, Fe_3_O_4_@COF@Au@PEI-GF, which is guanidino-functionalized and magnetic. This composite not only enriches phosphopeptides in human serum but also specifically traps exosomes in serum based on the force between the guanidino group and the exosomal phospholipid bilayer (as shown in Figure 8a). Immediately after, their group [73] proposed magnetic COF composites functionalized with flexible branched polymer-connected Ti^4+^ (mCOF@ε-PL@THBA-Ti^4+^). The surface of the magnetic composites was modified amino-rich ε-poly-L-lysine (ε-PL), as shown in Figure 8b, which enhances the hydrophilicity of the material and reduces the nonspecific enrichment of non-phosphopeptides. 2,3,4-trihydroxybenzaldehyde (THBA) was used to modify the material’s surface; this was achieved by reacting the amino group with the aldehyde group to provide binding sites for Ti^4+^. The resulting material has a low detection limit (2 f mol for β-casein digest), high selectivity (molar ratio of β-casein: BAS was 1:5000), and high loading capacity (66.7 mg/g). The material was able to detect 16 endogenous phosphopeptides in healthy human saliva.

However, the multistep modification process on the material’s surface introduces significant spatial site resistance, and the cumbersome synthesis steps have an impact on the material. Therefore, there is a need to develop a simple and more efficient magnetic composite. He et al. [70] reported a one-pot synthesis of Fe_3_O_4_@TAPTDHTA-Ti^4+^ magnetic composites with inherent bifunctional groups (Tz and OH) based on tpt and DHTA. The composites have a large number of binding sites for Ti^4+^ due to their surface-abundant o-phenol hydroxyls, and a limit of detection as low as 0.05 f mol μL^−1^ (β-casein digest). The synthesis is shown in Figure 7(2a–2d) and Figure 8c. Additionally, the study detected 9, 4, and 12 enriched phosphopeptides in milk, human serum, and human salivary fluid, respectively. In the HeLa cell lyses, the study detected 3333 phosphopeptides, of which 3180 (95.4%) were monophosphopeptides and 153 (4.6%) were polyphosphopeptides. Ding et al. [74] applied a layer of COFs to Fe_3_O_4_@SiO_2_ microspheres through the condensation reaction between mesitylene triphenol (Tp) and 2-nitro-1,4-phenylenediamine (Pa-NO_2_) under vacuum and acetic acid-catalyzed conditions. Nitroamidation and phosphorylation were performed in an aqueous titanium sulfate solution, resulting in Fe_3_O_4_@SiO_2_@TpPa-Ti^4+^ (as shown in Figure 8d). The COFs layer’s surface area and abundant functional groups contributed to a Ti^4+^ loading of up to 14% (wt%). The binding capacity for phosphopeptides was up to 200 mg/g, with a low detection limit of 0.2 f·mol·μL^−1^ (α-casein digest) and a good selectivity (1:5000, α-casein to BAS molar ratio). In skimmed milk, 33 phosphopeptides were enriched, while in human serum, 4 phosphopeptides were enriched. In HeLa cell lysates, 3010 phosphopeptides were identified, of which 74.8% were monophosphorylated and 25.2% were polyphosphorylated. In rat brain lysates, 1084 phosphorylated peptides were detected.

### 4.3. Magnetic MOF Materials for Phosphopeptids Enrichment

**Figure 9:**
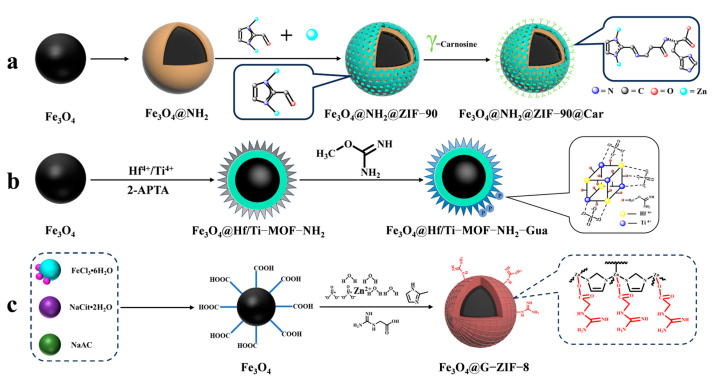
Magnetic metal–organic framework materials for enrichment of phosphopeptides, the synthesis steps of Fe_3_O_4_@NH_2_@ZIF−90@Car, Fe_3_O_4_@Hf/Ti−MOF−NH_2_−Gua and Fe_3_O_4_@G−ZIF−8 are represented by processes (**a**), (**b**), and (**c**), respectively.

To enrich phosphopeptides, Qi et al. [71] synthesized ZIF-90 on an aminated Fe_3_O_4_ core using an in situ method. They then utilized the aldehyde group in ZIF-90 to modify the carnosine peptide (Car) on the material’s surface through imine condensation, resulting in Fe_3_O_4_@NH_2_@ZIF-90@Car (shown in Figure 7(3a–3d) and Figure 9a). The phosphopeptide can be adsorbed synergistically by both the Zn^2+^ in ZIF-90 and the imidazole in Car. Under acidic conditions, imidazole is protonated, resulting in a positive charge on Fe_3_O_4_@NH_2_@ZIF-90@Car. This positive charge allows it to bind to the negatively charged phosphopeptide. Upon exposure to alkaline conditions, Fe_3_O_4_@NH_2_@ZIF-90@Car loses its proton and separates from the phosphopeptide. The Fe_3_O_4_@NH_2_@ZIF-90@Car composite exhibits a low detection limit of 0.1 f mol for the β-casein digest, a high selectivity with a 1:500 mass ratio for the tryptophan-digested mixture of β-casein and bovine serum albumin, and a high size exclusion with a 1:1000 mass ratio for β-casein digest to BSA. These properties are attributed to the regularly ordered pores of the MOFs, resulting in the detection of 17 phosphopeptides in skim milk and 28 phosphopeptides in human saliva.

Cao et al. [75] synthesized magnetic MOF materials modified with iron and titanium using the bimetallic ion-IMAC strategy. The resulting material, Fe_3_O_4_@MIL(Fe/Ti), was used for phosphopeptide enrichment in complex samples. Li et al. [76] modified guanidinium groups on the surface of magnetic MOF composites using the bimetallic ion IMAC strategy to obtain Fe_3_O_4_@Hf/Ti-MOF-Gua (shown in Figure 9b). This modification uses multiple affinity strategies to synergistically enrich phosphopeptides, increasing the phosphopeptide selectivity in complex samples to up to 1: 2000: 2000 (α/β-casein digest–α-casein–BSA). Zhang et al. [77] developed a one-step epitaxial growth strategy to prepare magnetic guanidinium-based functionalized MOF materials with multi-affinity sites (Fe_3_O_4_@G-ZIF-8) for the enrichment of global phosphopeptides. This approach was taken due to the potential for molecules derived from MOFs to clog the MOF’s pores and the relatively cumbersome synthesis process. Figure 9c shows the results of this strategy. The MOF layer uses an IMAC-based strategy with Zn^2+^ and a non-covalent strategy with guanidinium groups to interact with phosphopeptides. This multi-affinity site and strategy effectively enriches phosphopeptides in biological samples, demonstrating good selectivity and an excellent sensitivity of 1 f mol for β-casein digest. Using MALDI-TOF mass spectrometry, they detected 4 and 30 phosphopeptides in human serum and human saliva, respectively. Additionally, nano-lc-MS/MS detected 25 phosphopeptides in human serum and 14 in human saliva. Table 2 presents the properties of the core-shell magnetic mesoporous materials discussed in the text, which are employed for the enrichment of phosphopeptides.

In summary, the principle of phosphopeptide enrichment is based on the electrostatic interaction force between phosphopeptides and metal ions. The specific adsorption capacity of phosphopeptides can be enhanced by using high-valence metal ions (oxides) with stronger electrostatic interactions or bimetallic ions (oxides). The desired effect can be achieved by using mesoporous shells, such as COFs, which have a larger specific surface area or a larger number of fixed sites for the metal ions. To achieve synergistic adsorption of phosphopeptides, a variety of processes and strategies can be used, such as using imidazole or guanidinium. It is important to pay attention to their spatial site resistance to prevent clogging of the pores and to maintain a high adsorption efficiency.

## 5. Conclusions and Perspectives

Post-translationally modified peptides, such as glycopeptides and phosphopeptides, are closely related to human health. Enriching and identifying these peptides are of great significance. This paper summarizes the research progress for new magnetic mesoporous materials in enriching glycopeptides and phosphopeptides in the last five years. The majority of strategies for enriching glycopeptides involve modifying hydrophilic compounds. This is because the most significant distinction between glycopeptides and other endogenous peptides is that the glycosyl portion of glycopeptides is hydrophilic. In comparison to conventional mesoporous silica, COFs and MOFs are formed by covalent linkage between organic ligands or ligand linkage with metal ions, which results in a large number of sites for binding hydrophilic compounds on the surface of COFs and MOFs. When biomarkers for various diseases are explored in the future, magnetic COF and MOF materials can be targeted for self-assembly based on the characteristics of these disease markers, and faster and more accurate disease diagnosis methods can be developed.

For phosphopeptides, the majority of current enrichment strategies are based on IMAC and MOAC. However, there are various phosphopeptides with different characteristics in real samples, and a single IMAC or MOAC strategy cannot comprehensively enrich all phosphopeptides in real samples. Consequently, the majority of phosphopeptide enrichment strategies are based on multiple affinity strategies, which can compensate for the shortcomings of a single strategy. However, the use of multiple enrichment strategies simultaneously necessitates multi-step modification during the synthesis of the enriched materials. In contrast, MOFs exhibit advantages that are not comparable to mesoporous silica and COFs due to their inherent metal ions. Consequently, the simplification of the synthesis steps of magnetic mesoporous materials and the development of high-performance adsorbent materials represent dominant research focuses for the near future. Furthermore, the kinetic mechanism of the adsorption of metal oxides on phosphopeptides remains poorly understood. To gain deeper insights into the pathogenic mechanisms of aberrant post-translationally modified phosphopeptides, it is essential to enhance the investigation of the adsorption mechanism of metal oxides on phosphopeptides. The development of material science has led to the discovery of new functionalized magnetic mesoporous materials and enrichment strategies. These advancements provide new ideas for the enrichment of post-translationally modified peptides. This will play a crucial role in the discovery of disease biomarkers based on post-translationally modified peptidomics.

## Figures and Tables

**Table 1:** A representative selection of magnetic mesoporous materials that have been employed in the enrichment of glycopeptides over the past five years.

Material	Functional Part	Limit of Detection (HRP Digest Solution)	Real Sample	Glycopeptides	Glycoproteins	Ref.
L−Cys−Fe_3_O_4_@mSiO_2_	L-Cys	1 f·mol·μL^−1^	Healthy human saliva Gastric cancer patient saliva	46, 36		[46]
Fe_3_O_4_@mSiO_2_−TSG	N-(3-triethoxysilylpropyl) gluconamide	0.1 f·mol·μL^−1^	Healthy human serum Breast cancer serum	162 156	71 70	[47]
Fe_3_O_4_−CG@mSiO_2_	cysteine and glutathione	5 a mol·μL^−1^	Human serum exosomes	156	64	[48]
mCTpBD	cysteine and glutathione	0.5 f·mol·μL^−1^	Saliva of healthy people; saliva of patients with inflammatory bowel disease	28, 32, 49 27, 39, 40		[53]
Fe_3_O_4_@TpBD@Au@GSH	glutathione	0.1 f·mol·μL^−1^	5 μL of human serum and human saliva	492 160	134 64	[49]
magMOF@Au−maltose	maltose	0.1 f·mol·μL^−1^	1 μL of human serum	113	46	[54]
mMOF@Au−MSA	mercaptosuccinic acid	0.5 f·mol·μL^−1^	2 μL of serum from breast cancer patients	307	96	[55]
MagZr−MOF@G6P	glucose-6-phosphate	0.1 f·mol·μL^−1^	Urine of kidney cancer patients and healthy people	123 111	78 76	[50]

**Table 2:** A representative selection of magnetic mesoporous materials that have been employed in the enrichment of phosphopeptides over the past five years.

Material	Functional Part	Limit of Detection (β-Casein Digests)	Real Sample	Phosphopeptide	Ref.
Fe_3_O_4_@mSiO_2_−Ti^4+^	Ti^4+^	0.1 f·mol·μL^−1^	healthy human serum and saliva	4, 13	[61]
Fe_3_O_4_@mSiO_2_−PO_3_−Ti^4+^/Zr^4+^	PO3−Ti^4+^/Zr^4+^	1 f·mol·μL^−1^	healthy saliva and enteritis saliva	25, 85	[64]
Fe_3_O_4_@mSiO_2_@APA@Ti^4+^/Nb^5+^	APA@Ti^4+^/Nb^5+^	0.05 f·mol·μL^−1^	human serum and saliva	4, 24	[65]
Fe_3_O_4_@mSiO_2_@Ti^4+^−Zr^4+^	PDA@Ti^4+^Zr^4+^	0.1 f·mol·μL^−1^	human saliva	104	[66]
Fe_3_O_4_@nSiO_2_@mSiO_2_@TiO_2_	TiO_2_	0.5 f·mol·μL^−1^	nonfat milk and HepG2 cells	13, 1904	[68]
Fe_3_O_4_@TiO_2_−ZrO_2_@mSiO_2_	TiO_2_−ZrO_2_	0.2 f·mol·μL^−1^	human saliva	30	[69]
Fe_3_O_4_@COF@Au@PEI−GF	Polyethyleneimine, guanidyl	0.02 f·mol	human serum	4	[72]
mCOF@ε−PL@THBA−Ti^4+^	Ti^4+^	2 f·mol	healthy human saliva	16	[73]
Fe_3_O_4_@TAPTDHTA−Ti^4+^	Ti^4+^	0.05 f·mol·μL^−1^	nonfat milk, human serum, human saliva and HeLa cell lysate	9, 4, 12, 3333	[70]
Fe_3_O_4_@SiO_2_@TpPa−Ti^4+^	Ti^4+^	0.2 f·mol·μL^−1^	non-fat milk, human serum, HeLa cell lysates and rat brain lysates	33, 4, 3010, 1084	[74]
Fe_3_O_4_@NH_2_@ZIF-90@Car	carnosine	0.1 f·mol	nonfat milk and human saliva	17, 28	[71]
Fe_3_O_4_@G-ZIF-8	guanidyl	1 f·mol	human serum and saliva MALDI-TOF MS nano-LC-MS/MS	4, 30, 25, 14	[77]

## Data Availability

No new data were created or analyzed in this study. Data sharing is not applicable to this article.
